# The Gut-Lung Axis in Cystic Fibrosis

**DOI:** 10.1128/JB.00311-21

**Published:** 2021-09-23

**Authors:** Courtney E. Price, George A. O’Toole

**Affiliations:** a Department of Microbiology and Immunology, Geisel School of Medicine at Dartmouth, Hanover New Hampshire, USA; McGovern Medical School

**Keywords:** cystic fibrosis, intestine, microbiota, inflammation, gut-lung axis

## Abstract

Cystic fibrosis (CF) is a heritable, multiorgan disease that impacts all tissues that normally express cystic fibrosis transmembrane conductance regulator (CFTR) protein. While the importance of the airway microbiota has long been recognized, the intestinal microbiota has only recently been recognized as an important player in both intestinal and lung health outcomes for persons with CF (pwCF). Here, we summarize current literature related to the gut-lung axis in CF, with a particular focus on three key ideas: (i) mechanisms through which microbes influence the gut-lung axis, (ii) drivers of microbiota alterations, and (iii) the potential for intestinal microbiota remediation.

## INTRODUCTION

Cystic fibrosis (CF) is a heritable, autosomal recessive disease that affects more than 70,000 people worldwide (1). CF is caused by mutations in the cystic fibrosis transmembrane conductance regulator (*CFTR*) gene, which leads to altered secretion of chloride and bicarbonate, and buildup of abnormally thick mucus in the lungs and intestinal tract (1–4). Recent microbiological studies have focused heavily on the lungs of persons with CF (pwCF), due to the fact that lung complications are currently the most common cause of early mortality (5–7). Environmental changes in the lung, including decreased mucociliary clearance, aberrant immune function, and chronic inflammation allow opportunistic pathogens to chronically colonize the lungs of pwCF (8–11). The opportunistic pathogens in the CF airway include polymicrobial communities of bacteria, fungi, and viruses, all of which have the potential to interact with each other, impact patient health outcomes, and interfere with antibiotic treatment (12–17). The CF lung microbiota has been extensively studied and reviewed and will not be addressed in depth here (6, 10, 15–19).

The defect in the *CFTR* gene causes alterations to the intestinal tract, both inherently and via clinical treatments used to alleviate symptoms of CF (2, 3, 20). In the intestinal tract, the CFTR defect alters bile acid production, diminishes secretion of pancreatic enzymes, and reduces bicarbonate secretion, all of which lead to both a more acidic intestinal pH and higher fecal fat content (2, 20, 21). pwCF can experience intestinal blockages at birth (meconium ileus), small intestinal bacterial overgrowth (SIBO), distal intestinal obstruction syndrome (DIOS), inflammation, and dysmotility, and pwCF often struggle with sufficient weight gain in early childhood due to fat malabsorption (2, 22). Malnutrition was a significant contributor to early mortality of pwCF in the mid-20th century, but clinical interventions such as pancreatic enzyme replacement therapies and appropriate dietary interventions have significantly improved patient outcomes (23). Clinical interventions that improve patient outcomes can also alter the intestinal microbiota. Such interventions include frequent antibiotic treatment for respiratory exacerbations, altered diet, pancreatic enzyme replacement, and CFTR modulator therapies. Different interventions have the potential to either positively or negatively impact the gut microbiota, but even with interventions that improve intestinal outcomes, such as CFTR-targeted modulator therapies, the gut microbiota of pwCF remains distinct from healthy controls (24–26). Furthermore, with the advent of modulators, CF patients are living longer and healthier lives. These new medications are a huge advancement, but have also revealed intestinal complications later in life, including higher rates of inflammatory bowel disease (IBD) and colon cancer in pwCF (27–30). There is a critical need to understand the gut microbiota of pwCF and the role these microbes plays in disease progression, not only in the intestine but system wide.

Several studies have documented alterations in the gut microbiota of pwCF (31–35). Methods have evolved rapidly over the last several years, with early studies using culture, quantitative reverse transcriptase PCR (qRT-PCR), or denaturing gradient gel electrophoresis (DGGE), while later studies have used 16S rRNA gene amplicon sequencing or shotgun metagenomics to determine what microbes are present, how the relative abundance of specific microbes are altered compared to healthy populations, and how the changing suite of microbes can alter functional capacities of the microbiota. Efforts have also been made within cohorts of pwCF to associate overall intestinal microbiota community structure, the presence of specific microbes, and the inflammatory status of the intestine with various health outcomes.

The “gut-lung axis” is an emerging concept linking the state of the gut microbiota to respiratory health outcomes (37). The gut microbiota has been suggested to influence lung health outcomes in various diseases, including asthma, chronic obstructive pulmonary disease (COPD), and CF (37). As correlative clinical data linking the microbiota to metrics of lung health have become more available, new research has just begun to focus on immunological and microbiological mechanisms driving the gut-lung connection(s) in CF. The gut-lung axis has been reviewed extensively; most of the studies to date focus on either a breadth of respiratory diseases or focus primarily on the respiratory microbiota in pwCF (37–48). CF is distinct from other instances of the gut-lung axis because this disease has direct manifestations in multiple organs, making it more difficult to disentangle cause and effect. Here, we comprehensively review the gut-lung axis in CF with a specific focus on dysbiosis, clinical outcomes, mechanistic insights, and potential therapies for pwCF.

## WHAT IS THE GUT-LUNG AXIS?

The mammalian gut microbiota is known to affect the chemistry and/or function of all other organs in the body (49). Here, we define the gut-lung axis as the ability of the gut microbiota to influence the course or outcome of underlying lung disease, and vice versa. There is strong evidence for the existence of the gut-lung axis in asthma, and emerging evidence in other respiratory diseases, including COPD and CF (37–48) (Table 1). The gut-lung axis is thought to be driven primarily by immunological cross talk between the distal but similarly structured mucosal environments of the lung and the intestinal tract. There is also evidence of direct transmission of microbes and metabolic cross talk between these organ systems, as described below. In all cases, the mechanism of how intestinal microbiota effect lung health outcomes is complex and not yet fully elucidated.

**TABLE 1:** Original publications on the gut-lung axis in cystic fibrosis

Reference	Major clinical outcome(S)	Mechanism(S) or taxa of interest^*a*^
Human gut-lung axis		
Madan et al., 2012 (32)	Pathogens colonize the gut prior to the respiratory tract. The lungs and gut share a core microbiota.	Direction transmission from intestine. The gut and lung core microbiota is comprised of Streptococcus and *Veillonella.*
Hoen et al., 2015 (31)	Gut microbiota, but not oropharyngeal microbiota, composition during the first 6 mo of life is significantly different for infants who do vs do not experience respiratory exacerbation. Gut alpha-diversity positively correlates with longer time to initial exacerbation.	Intestinal *Parabacteroides* significantly decreased prior to Pseudomonas colonization. The gut and lung core microbiota comprised of Streptococcus*, Veillonella, Bifidobacterium, Clostridium, Blautia, Coprococcus*, and *Bacteroides.*
Antosca et al., 2019 (33)	Gut microbiota composition is significantly different for pwCF who have an exacerbation during the first yr of life.	*Bacteroides* is significantly decreased in infants with CF in the first yr of life, and *Bacteroides* and its products downregulate the proinflammatory cytokine IL-8.
Burke et al., 2017 (53)	Alpha-diversity positively correlates with ppFEV_1_.	*Dorea, Pseudobutyrivibrio*, and *Roseburia* significantly increased in adults with high ppFEV_1_.
Coffey et al., 2019 (54)	Intestinal taxa are significantly associated with ppFEV_1_. No correlation between alpha-diversity and ppFEV_1_.	*Adlercreutzia, Ruminococcaceae, Lachnospiraceae, Tyzzerella*, and *Candidatus Soleaferrea* positively correlate with ppFEV_1_.
Vanstone et al., 2015 (55)	Serum vitamin D, a product of microbial metabolism, is a significant predictor of annual no. of exacerbations.	
Jafari et al., 2013 (57)	Probiotic treatment significantly reduced exacerbation rate.	Commercial probiotic contained Lactobacillus casei, Lactobacillus rhamnosus, Streptococcus thermophilus*, Bifidobacterium breve*, Lactobacillus acidophilus, Bifidobacterium infantis, and Lactobacillus bulgaricus.
Loman et al., 2020 (58)	Secondhand smoke exposure alters the structure of the gut microbiota in children with CF.	Secondhand smoke exposure was associated with increased *Akkermansia*, Acinetobacter, and alpha-diversity. *Bifidobacterium* and *Lactobacillus* were decreased.
Caparrós-Martín et al., 2020 (96)	Bronchoalveolar lavage fluid from pwCF contains bile acid. Lung structural deterioration was greater in patients with high bile acid at follow-up.	Patients with high bile acid had significantly increased IL-1β and IL-6 but no increase in IL-8 or neutrophil elastase.
Flynn et al., 2020 (97)	The presence of bile acids in bronchoalveolar lavage fluid altered the lung microbiota composition in pwCF.	Pathogen counts were positively associated with bile acid quantity.
Schnapp et al., 2019 (105)	Fecal calprotectin decreases after antibiotic treatment for respiratory exacerbation.	
Bruzzese et al., 2007 (166)	Probiotic decreased respiratory exacerbations and hospitalizations, and increased body wt and ppFEV_1_.	
Dhaliwal et al., 2020 (116)	Intestinal inflammation is linked to decreased growth but not ppFEV_1_.	
Mechanistic immunology studies		
Bazett et al., 2016 (56)	Streptomycin antibiotic treatment reduced airway hyperresponsiveness in CF mice. Streptomycin-treated mice had reduced IL-17^+^ γδ T cells and increased Th17 (CD4^+^ IL-17^+^), CD8^+^ IL-17^+^, and CD8^+^ IFNγ^+^ T-cells.	
Hiippala et al., 2020 (109)	*Odoribacter* promotes IL-10 production by PBMCs, and inhibits IL-8 secretion by gut epithelial cells	*Odoribacter* associated with positive airway outcomes in other studies
Ikpa et al., 2020 (110)	Increased transcriptional activation of innate and adaptive immune responses in the ileum of CF mice is reversible by antibiotic treatment.	
De Lisle, 2016 (111)	The CF intestinal lumen has reduced enterocyte maturation, evidenced by reduced expression of enterocyte genes and proteins. Enterocyte maturation can be restored with laxative but not antibiotic treatment.	
Meeker et al., 2020 (36)	Germfree CF, wild-type, and heterozygote mice were colonized with stool from wild-type mice. The microbiota of CF and wild-type mice were significantly different. Microbial colonization increased TH17 in CF mice relative to wild-type.	CF mice had reduced alpha-diversity, reduced *Parabacteroides* and *Lachnoclostridium*, and increased Escherichia.

a We note shown/predicted mechanism or associated microbiota, when assessed in the publication.

Early evidence of the existence of the gut-lung axis for pwCF demonstrated that common lung pathogens colonize the gut prior to colonization of the upper respiratory tract (URT) and that when microbes are increasing over time in the gut, they tend also to increase over time in the URT; this is also true for microbes that are decreasing in abundance (32). Two different clinical outcomes are typically used to assess lung health in pwCF: (i) percent predicted forced expiratory volume in 1 s (ppFEV_1_) and (ii) respiratory exacerbations, which represent a nonstandardized metric of worsening CF symptoms that often require antibiotic treatment and even hospitalizations (50–52). These metrics of lung health have been used to examine the relationship between the intestinal microbiota and the lungs in pwCF. For example, two studies demonstrated that the composition of the gut microbiota of children with CF (cwCF) who have had a respiratory exacerbation is significantly different from those without exacerbation in the first 6 to 12 months of life (31, 33). Furthermore, intestinal microbiota alpha-diversity, a measure that encompasses both microbial richness and evenness, is also positively correlated with increased time to initial exacerbation in infants, and higher ppFEV_1_ in adults (31, 53). Interestingly, there was not a significant association between the URT microbiota composition and airway exacerbation in infants in the one study where this association was investigated (31), indicating that the intestinal microbiota may play a larger role in predicting lung health outcomes than airway microbes, particularly early in life.

Finally, alterations of individual genera in the gut microbiota may also predict events in the airway, as *Parabacteroides* was significantly decreased in the gut prior to initial Pseudomonas colonization in a cohort of infants with CF (31). There are also positive correlations between specific intestinal genera (i.e., *Adlercreutzia*, *Ruminococcaceae* strain NK4A214, *Lachnospiraceae* strain NC2004, *Tyzzerella* sp. strain 3, and "*Candidatus* Soleaferrea") and ppFEV_1_ in children, and a significant increase in relative abundance of genera (i.e., *Dorea*, *Pseudobutyrivibrio*, and *Roseburia*) in adults with high ppFEV_1_ (53, 54). Interestingly, these increased taxa do not overlap between the adult and child cohorts, and it is unclear whether this observation is a true age-dependent difference in the microbiota composition or due to study-specific differences. Dietary factors, such as serum vitamin D, are also predictors of a reduced annual number of exacerbations (55). In CF mice, airway hyperresponsiveness, which was tested by methacholine challenge and, therefore, in the absence of pathogens in the lungs, was reduced when the mouse gut microbiota was altered by streptomycin treatment (56). These results indicate that specific gut symbionts and/or the metabolites they produce can play a role in altering lung health outcomes. It may also be possible to remediate the gut microbiota of pwCF to positively impact lung health outcomes; in one study, a probiotic treatment was shown to reduce exacerbation rates (57).

It is important to note the potential for gut-lung axis bidirectionality. That is, there is evidence that the gut microbiota influences lung health outcomes, but it is also possible that the lung influences gut microbiota structure and the intestinal inflammatory state. In CF, this notion is highlighted by a study demonstrating that secondhand smoke can alter the gut microbiota of pwCF (58). In this review, we address microbial alterations in the context of CF, with a focus on how intestinal microbes may influence lung health outcomes through a variety of mechanisms. We also examine how underlying CFTR dysfunction and typical treatment regimens for pwCF both contribute to microbial alterations. Finally, we examine emerging methods for remediating the gut microbiota.

## MICROBIAL DYSBIOSIS: AN OVERVIEW

A small but growing number of studies have addressed the microbial structure of the CF intestinal microbiota in both children and adults (Table 2). The microbiota structure of pwCF is consistently significantly altered relative to non-CF cohorts across studies with different methods, patient ages, and countries of origin. Alpha-diversity is consistently decreased in the stool microbiota of pwCF relative to healthy controls (33, 35, 59–63).

**TABLE 2:** Summary of intestinal microbiota studies in CF

Paper	Study type	No. of subjects	Age(s)	Method(s)	Sample	Age, temporal, or health component(s)
Duytschaever et al., 2011 (176)	Cross-sectional and longitudinal, healthy sibling comparison	21 CF/24 HC (cross-sectional), 2 family units (longitudinal)	9 mo–15 yrs	Culture, DGGE	Stool	Lower temporal stability in CF in longitudinal portion of study.
Duytschaever et al., 2013 (177)	Cross-sectional and longitudinal, healthy sibling comparison	21 family units (cross-sectional), 9 family units (longitudinal)	0.8–15.7 yrs	Culture, DGGE, RT-PCR	Stool	*Clostridium* is positively associated with ht and wt.
Lynch et al., 2013 (89)	Mouse CFTR^−/−^	*n* = 3 per condition	6 wks	Microarray	Ileal	CF microbiota had reduced variability.
del Campo et al., 2014 (71)	Probiotic clinical trial prospective double-blind crossover	30 CF	8–44 yrs	16S	Stool	Probiotic treatment with L. reuteri improved gastrointestinal quality of life scores, decreased calprotectin, and reduced proteobacteria.
Hoffman et al., 2014 (34)	Longitudinal, age matched	12 pwCF, 12 HC	Infants to 5 yrs	Metagenomics	Stool	E. coli is increased in children with CF.
Nielsen et al., 2016 (59)	Cross-sectional, age matched	23 CF, 35 HC	0.87–17 yrs	16S	Stool	Greater variability in CF cohorts. Pancreatic sufficiency trended towards increased diversity.
Vernocchi et al., 2018 (67)	Cross-sectional, age matched	28 CF, 31 HC	1–6 yrs	16S	Stool	Younger healthy controls clustered more closely with CF.
Bazett et al., 2016 (56)	Mouse BALB/c *Cftr* null	*n* = 8–13 per group	>3 –12 wks	16S	Small intestine	Streptomycin reduces airway hypersensitivity.
Debyser et al., 2016 (79)	Cross-sectional, healthy sibling comparison	15 CF, 15 HC	1.6–15.6 yrs	Proteomics	Stool	Intestinal microbiota alterations associated with pwCF are detectable by shotgun proteomics. Calprotectin is not altered in CF stool; both other inflammatory markers are increased.
Manor et al., 2016 (35)	Longitudinal, age matched	14 CF, 12 HC	15 days–5 yrs	Metagenomic	Stool	Microbiota composition outcomes are not driven by recent antibiotics or breastfeeding. Proteobacteria, driven by E. coli, are increased in pwCF.
Al-Momani et al., 2016 (88)	Cross-sectional	15 CF, 14 HC	Adults	16S	Gastric juice	Intestinal microbiota alterations associated with pwCF are also apparent in gastric juice.
Fouhy et al., 2017 (66)	Cross-sectional	6 CF, 6 HC	Adults	Metagenomic	Stool	Intestinal microbiota functionality is altered for pwCF.
Burke et al., 2017 (68)	Cross-sectional	60 CF, 99 HC	Adults	Culture	Stool	C. difficile carriage is asymptomatic and not correlated with clinical outcomes.
Burke et al., 2017 (53)	Cross-sectional	43 CF, 69 HC	Adults	16S	Stool	CF and therapies have a larger impact than specific treatments (proton pump inhibitors, antibiotics).
Miragoli et al., 2017 (120)	Cross-sectional	30 CF	Adolescents, 10–22 yrs	PCR-DGGE and qPCR	Stool	Microbiota of pwCF homozygous vs heterozygous for dF508 was not significantly different. Archaea were detected at lower rates in CF.
de Freitas et al., 2018 (62)	Cross-sectional	19 CF, 17 HC	Children with median ages 3 and 4 yrs	FISH	Stool	Antibiotic exposure reduced *Bifidobacterium* within the CF cohort.
Wang et al., 2019 (60)	Cross-sectional	19 CF, 16 HC	19–55 yrs	16S	Stool	The CF microbiota retains the ability to ferment HAMS but does so with a different suite of microbes.
Antosca et al., 2019 (33)	Longitudinal	21 CF, 409 HC	6 wks–12 mo	16S	Stool	IL-8, β-diversity associated with airway exacerbation.
Coffey et al., 2019 (54)	Cross-sectional, age matched	27 CF/27 HC	0.8–18 yrs	16S	Stool	SCFA catabolism was increased in pwCF. Specific genera were positively correlated with ppFEV_1_ and growth.
Dayama et al., 2020 (87)	Cross-sectional	18 CF/15 HC	Adults	16S	Colonic mucosa	Bacterial toxins are enriched in pwCF. Several stool-associated microbial alterations (increased *Vellionella* and *Streptococcaceae*, decreased *Ruminococcaceae*) are consistent at the colonic mucosa.
Hayden et al., 2020 (61)	Longitudinal	207 CF/25 HC	3–12 mo	Metagenomic	Stool	Proteobacteria decreased over yr 1 for healthy but not CF, and increase is driven by E. coli in CF. H_2_ blockers increase E. coli. Age analysis indicates delayed maturation.
Loman et al., 2020 (58)	Cross-sectional	20 CF	3 mo–5 yrs	16S	Stool	Recent antibiotic exposure associated with decreased *Bacteroides*. Secondhand smoke exposure associated with increased Acinetobacter and *Akkermansia.*
Meeker et al., 2020 (36)	Germfree B6 Cftr^tm1Unc^ mice	7 CFTR^−/−^ 4 CFTR^+/+^ 4 heterozygous	1 yr	16S	Stool	CFTR mutant mice had altered microbiota and adaptive immune response. CF mice have increased TH17^+^ cells in the mesenteric lymph nodes and spleen.
Kristensen, 2020 (63)	Longitudinal	20 CF/45 HC	Infants up to 18 mo	16S	Stool	Reduced compositional stability in CF.

**FIG 1:**
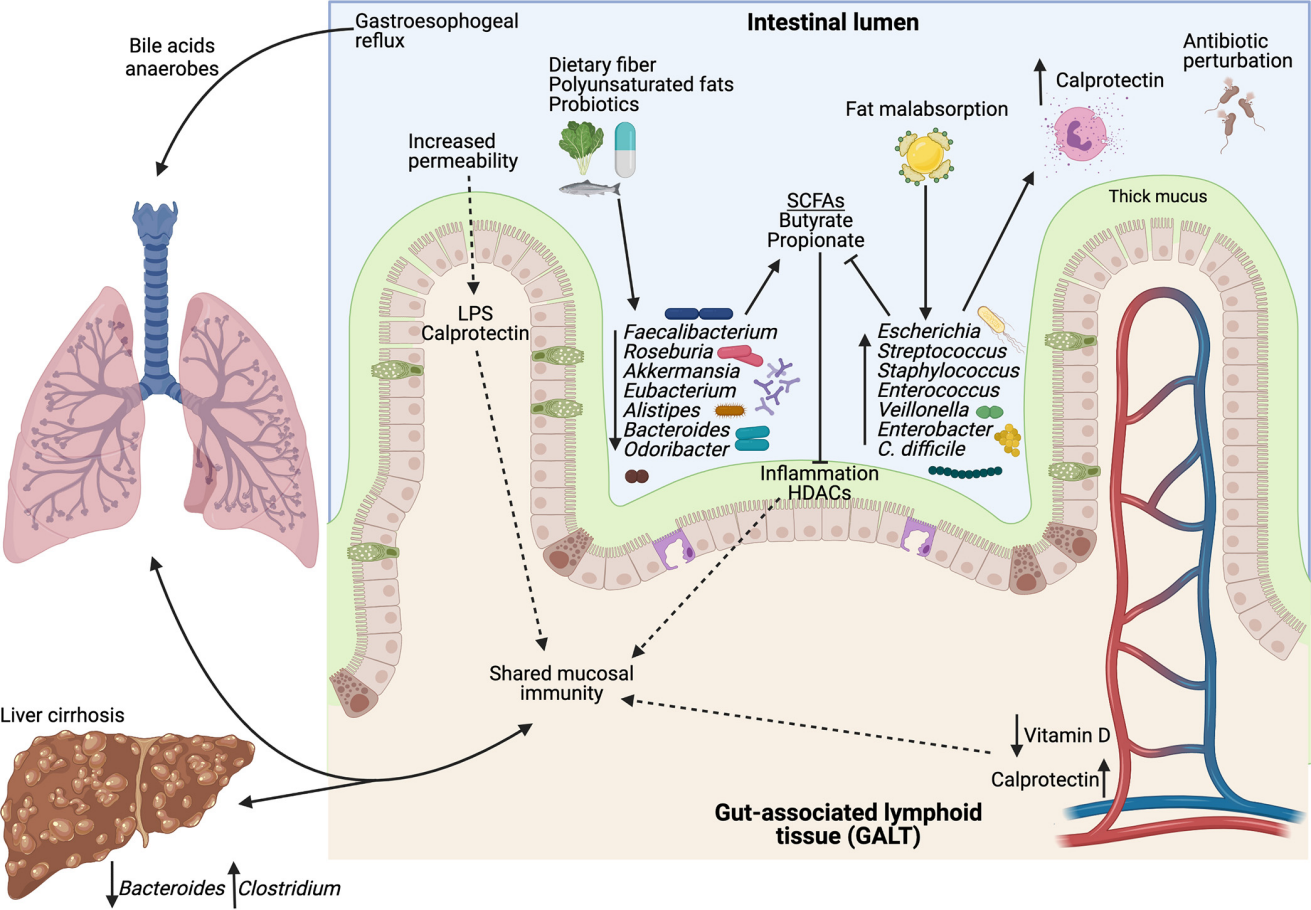
The gut-lung axis in cystic fibrosis. A summary of the alterations commonly found in the intestinal tract of pwCF and how these changes may affect lung and liver health outcomes. CF is associated with decreases in beneficial, SCFA-producing microbes (i.e., *Faecalibacterium*, *Roseburia*, *Akkermansia*, *Eubacterium*, *Alistipes*, and *Odoribacter*) and increases in SCFA-degrading, potentially pathogenic taxa (i.e., Escherichia, Streptococcus, Staphylococcus, *Enterococcus*, *Veillonella*, Enterobacter, and C. difficile). Microbial alterations are driven in part by higher fecal fat content, frequent antibiotic treatment, and thick intestinal mucus. These alterations lead to a high inflammatory environment that affects systemic and lung health outcomes through shared mucosal immunity and direct transmission of intestinal contents to the lungs. Liver cirrhosis is associated with decreased *Bacteroides* and increased *Clostridium* and may also be driven by shared mucosal immunity. The figure was created with BioRender.com.

Alterations in specific taxa can vary depending on the cohort examined, but several consistencies have emerged (Table 3; Fig. 1). These consistencies frequently encompass the expansion of known pathogens and the relative decrease of genera with beneficial members. For example, several studies have documented a relative increase of Escherichia coli, with this result being particularly frequent in studies focused on children and adolescents with CF (34, 35, 54, 59, 61, 63). Hayden et al. found a significant increase in *Proteobacteria* at the phylum level and that the expansion of *Proteobacteria* is driven by E. coli (61).

**TABLE 3:** Taxonomic alterations associated with the CF intestinal microbiota

Phylum^*a*^	Class	Order	Family	Genus	Species
↑*Proteobacteria* (35, 54, 61), ↓*Proteobacteria* (53, 66)	↑*Gammaproteobacteria* (54)	↑*Enterobacteriales* (54)	↑*Enterobacteriaceae* (54)	↑Escherichia*, Shigella* (54, 59, 63)	↑E. coli (34, 35)
				↑Enterobacter (54)	↑E. cloacae (35)
					
↓*Verrucomicrboia* (35, 53, 54)	↓*Verrucomicrobiae* (54)	↓*Verrucomicrobiales* (54)	↓*Verrucomicrobiaceae* (54)		
			*Akkermansiaceae*	↓*Akkermansia* (63)	↓*A. municiphila* (35)
					
↑ *Firmicutes* (53, 66), *Firmicutes* (ns) (61), ↓*Firmicutes* (35, 61)	↑*Negativicutes* (54)	↑*Veillonales* (*Selemonadales*) (54)	↑*Veillonellaceae* (54)	↑*Veillonella* (33, 54, 59, 63)	↑*V. parvula* (35, 60), ↑ *V. atypica* (35), ↑ *V. dispar* (35)
				↑*Megasphaera* (59)	↑*M. microuciformis* (35)
	*Clostridia*	↑*Clostridiales* (35), ↓*Clostridiales* (59)	↑*Clostridiaceae* (67)	↓*Clostridium* (59, 63), ↑*Clostridium* (59, 66, 67)	↑C. difficile (35, 67), ↑C. perfringens (35), ↑*C. hathewayi* (66), ↓*C. asparagiforme* (35), ↓*C. bartlettii* (35), ↓*Clostridium* cf (35), ↓*C. hathewayi* (35), ↓*C. leptum* (35), ↓*C. scindens* (35), ↑*C. symbiosum* (60), ↓*C. symbiosum* (35)
			↓*Lachnospiraceae* (59, 67)	↓*Roseburia* (33, 53, 59)	↓*R. intestinalis* (35), ↓*R. inulinivorans* (35)
				↓*Dorea* (67)	↓*D. formicagenerans* (66, 67)
				↑*Blautia* (59, 66)	↓*Blautia* (unclassified) (35)
				↓↑*Lachnospiraceae* (53, 59)	
			↓Ruminococcaceae (54)	↓*Faecalibacterium* (35, 53, 59, 66, 67)	↓*F. prausnitzii* (35, 60, 66, 67), ↓*Faecalibacterium* cf (35)
				↓*Ruminococcu* (54)	*↓R. bromii* (35, 60), *↓*R. albus (35), *↓R. lactaris* (35), *↓R. obeum* (35), ↑*R. gnavus* (60, 66), ↑ *R. torques* (66)
			*Eubacteriaceae*	↓*Eubacterium* (54, 67)	↓E. rectale (35, 60), ↓*E. eligens* (35), ↓*E. siraeum* (35), ↓*E. ventriosum* (35)
	↑ *Bacilli* (66)	↑*Lactobacillales* (35, 66)	↑*Enterococcaceae* (54, 66)	↑*Enterococcus* (53, 54, 59, 66)	↑E. faecalis (35, 60, 66), ↑E. faecium (35)
			*Streptococcaceae*	↑Streptococcus (35, 63)	↑S. mitis (35), ↑*S. parasanguinis* (35), ↑S. thermophilus (66)
		*Bacillales*	*Straphylococcaceae*	↑Staphylococcus (67)	
	*Erysipelotrichia*	↓*Erysipelotrichales*	↓*Erysipelotrichaceae* (59, 67)	↓*Erysipelotrichaceae* (54)	
				*Solobacterium*	↓*S. moorei* (67)
				*Erysipelatoclostridium (Clostridium)*	↑*E. innocuum* (54)
				*Holdemania*	↓*H. filiformis* (35)
			*Coprobacillaceae*	*Coprobacillus*	↓*C. bacterium* (35)
	*Tissierellia*	*Tissierellales*	*Peptoniphilace*	*Parvimonas*	↑P. micra (67)
					
↓*Bacteroidetes* (35, 53, 61, 66), *Bacteroidetes* (ns) (54)	*Bacteroidia*	*Bacteroidales*	↓*Rikenellaceae* (54)	↓*Alistipes* (54, 59)	↓*A. putredinis* (35, 66), ↓*A. shahii* (35)
			*Bacteroidaceae*	↓*Bacteroides* (33, 62), *Bacteroides* (ns) (59), ↑*Bacteroides* (53)	↓*B. caccae* (35), ↓*B. intestinalis* (35), ↓*B. ovatus* (35), ↓B. vulgatus (35), ↓*B. xylanisolvens* (35)
			↓*Prevotellaceae* (53)	↓*Prevotella* (53)	↓Prevotella copri (60)
			*Odoribacteraceae*	↓*Odoribacter* (53, 59)	
			*Barnesiellaceae*	↓*Barnesiella* (59, 66)	↓*B. intestinihominis* (66)
					
↑*Actinobacteria* (35, 53), *Actinobacteria* (ns) (61), ↓*Actinobacteria* (61, 66)	*Coriobacteria*	*Eggerthellales*	*Eggerthellaceae*	↓ *Eggerthella* (63)	↓*E. lenta* (35, 67), ↑*E. lenta* (66)
				*Gordonibacter*	↓*G. pamelaeae* (35)
		*Bifidobacteriales*	↓*Bifidobacteriaceae* (53)	↓*Bifidobacterium* (53, 66)	↓B. longum (66)
		*Actinomycetales*	*Actinomycetaceae*		↑Actinomyces odontolyticus (35)
			*Micrococcineae*		↑Rothia mucilaginosa (35)
		*Propionibacteriales*	*Propionibacteriaceae*	*Propionibacterium*	↑P. acnes (67)

a This table was generated from 12 studies comparing the CF versus non-CF stool microbiota using 16S or shotgun metagenomics sequencing. Lack of an arrow indicates that the given taxon was either not assessed or not significantly altered in any study. Nonsignificant results (ns) are noted only for taxa that are significantly altered in different directions in separate studies.

*Firmicutes* represent a major component of the gut microbiota (64, 65), with altered relative abundance in pwCF depending on the location, age, and health status of study participants. At the phylum level, results conflict as to whether pwCF have decreased, increased, or unchanged numbers of *Firmicutes* (Table 3). However, consistencies within the *Firmicutes* at the genus level indicate that pwCF show a decrease in beneficial, short-chain fatty acid (SCFA)-producing symbionts and an increase in potential pathogens. Particularly notable for pwCF is the decrease in the butyrate-producing symbionts Faecalibacterium prausnitzii and *Roseburia* (33, 35, 53, 59, 60, 63, 66, 67). The role of SCFA production by intestinal microbes in lung health outcomes is discussed in depth below in the section on microbial metabolism and immune regulation. In contrast, increases in the opportunistic pathogens *Enterococcus* and Clostridium difficile have frequently been documented in pwCF (33, 35, 54, 59, 60, 63, 66, 68). Interestingly, while pwCF have high carriage rates of C. difficile, they are typically asymptomatic for the acute intestinal complications associated with this microbe (68); C. difficile carriage in pwCF has been shown in a longitudinal study to be transient, with fluctuations in strain and amount present (69). pwCF also have higher relative abundance of *Veillonella*, a taxa that is increased in new-onset Crohn’s disease and is also detected in the lungs and URT of pwCF (18, 31, 32, 70). Microbes that are of concern for CF lung pathogenesis, including Streptococcus and Staphylococcus, have also been detected in the CF gut at higher rates or in a higher relative abundance (32, 35, 63, 66, 67). Extensive alterations within the *Firmicutes* families *Lachnospiraceae* and *Ruminococcaceae*, including alterations in *Blautia*, non-difficile *Clostridium*, *Ruminococcus*, and poorly described *Lachnospiraceae* species, have been documented, but both the cause and the impact of these changes are unclear.

At a broad taxonomic level, *Bacteroidetes* and *Verrucomicrobia* are consistently decreased in pwCF (35, 53, 54, 61, 66, 71). Within *Verrucomicrobia*, pwCF have decreased *Akkermansia* (35, 63), an intestinal symbiont associated with gut health that has anti-inflammatory properties and is known to contribute to gut barrier integrity (72–75). However, the benefits of *Akkermansia* may have contextual nuance, as this genus is more prevalent in the low-fiber, high-fat gut microbiota of persons from industrialized areas (76). The most prominent alterations associated with *Bacteroidetes* are decreases in *Alistipes*, *Bacteroides*, *Prevotella*, *Odoribacter*, and *Barnesiella*, all of which have been documented by multiple studies (Table 3). The effects of both *Alistipes* and *Bacteroides* on human health are controversial, with effects likely dependent on disease context and bacterial strains involved (77, 78). In one *in vitro* study, *Bacteroides* secreted products were demonstrated to decrease the production of proinflammatory cytokine interleukin 8 (IL-8) by CFTR^−/−^ gut epithelial cells (33). Additionally, while *Bacteroides* is decreased in most United States-based studies (33, 35), a large Irish study found that *Bacteroides* was actually increased in pwCF, while an Australian study found unaltered *Bacteroides* (52, 59). Age plays a key role in microbiota structure, and may explain these conflicting results, as each cohort varied greatly in age, except for the U.S. cohorts, which were both in infants. The known differences in treatment regimens between countries and even at the level of individual CF centers also raises the question of the extent to which *Bacteroides* relative abundance is influenced by treatment choices.

Proteomic studies, such as the one by Debyser et al., can give some insight into both microbiota community structure and function (79). In this study, stool was collected from children with and without CF, and shotgun proteomics was used to identify both human and microbial proteins in the stool. This is a novel approach in the CF field, and can also be used to infer relative abundances of microbes. The investigators found increased *Proteobacteria* and decreased *Firmicutes*, including *Faecalibacterium* and *Roseburia*, all of which have been described in 16S and metagenomic studies. Interestingly, these investigators saw an increase in proteins at the phylum level from *Bacteroidetes*, which is the opposite of what has been determined by sequencing studies. Further studies are needed to determine whether this difference is due to a true increase in *Bacteroidetes* abundance in this cohort, or a more nuanced answer such as higher activity of *Bacteroidetes* (or specific genera in this phylum) within the CF gut environment. This finding also highlights the importance of utilizing diverse approaches in analyzing the gut microbiota. This is not to suggest that increasingly complex technologies are always needed; basic methods such as qRT-PCR or culture can prove useful for analyzing absolute abundance of gut microbes and are rarely applied in CF gut microbiota studies. For example, Fouhy et al. utilized qRT-PCR to test microbial load in the stool of pwCF and concluded that, despite the fact that pwCF have SIBO, they do not have higher total bacterial loads in their stool (66).

The gut microbiota develops rapidly from infancy through early childhood, stabilizing between 3 and 5 years of age (80, 81). Maturation of the early gut microbiota is especially important for immune training and proper immune development (82–84). pwCF exhibit significantly altered gut microbiota structure, lower alpha-diversity, and lower stability in microbiota composition beginning in infancy (33, 54, 63). Depending on the study, alpha-diversity has been shown to increase either more slowly than healthy cohorts or not at all throughout the first year of life (33, 61). While alpha-diversity does increase modestly with age in pwCF, it remains lower throughout childhood and into adolescence (59). Unsurprisingly, some gut microbiota alterations are age-specific (59, 61) and may explain conflicting data between certain studies (Table 3). For example, Streptococcus abundance trends up over time in pwCF, but down for healthy controls (59). Interestingly, while pwCF have an altered gut microbiota at all ages, dysbiosis appears most striking in early life and decreases with age, particularly for E. coli-related dysbiosis (35, 61). Additionally, an age-based analysis by Hayden et al. indicates that maturation of the CF gut microbiota is delayed (61).

Most gut microbiota studies profile stool samples for bacterial relative abundance. Collection of stool samples is noninvasive, which is especially appealing when working with pwCF, who already have a high medical burden. However, there is scientific value in analyzing other types of intestinal samples because the gut microbiota has important spatial structure that is not captured by stool (85, 86). Dayama et al. examined mucosal biopsies from adults with CF obtained during surveillance colonoscopies and confirmed that several known alterations in stool are also consistent at the mucosa, including increases in *Veillonella* and *Streptococcaceae* and decreases in *Ruminoccocaceae* (87). However, they also identified novel alterations, including a decrease in the butyrate-producing genus *Butyricimonas*. A study of gastric juice in pwCF confirmed that specific alterations in stool also occur in these samples; *Bacteroides* and *Faecalibacterium* were both decreased (88).

Despite large differences in the human and mouse gut microbiota, the altered gut microbiota is recapitulated to some extent in mouse models; for example, CF mice have altered microbial structure and reduced diversity relative to wild-type mice (36, 56, 89). Specific alterations are study dependent, with one study in germfree mice recapitulating several aspects of the human microbiome, including increased Escherichia and decreased *Parabacteroides* and *Lachnoclostridium* (36). A separate study found that CF mice had decreased relative abundance of intestinal *Akkermansia* and increased *Clostridium* (56). A notable difference documented in the small intestine of mice was increased *Bacteroidaceae* (89), as represented by probes for B. fragilis. While specific alterations across studies were not largely consistent, one trend seen in two separate studies was an increase in *Lactobacillus* (36, 56), which may be a change that is specific to mice, as it is not seen in pwCF.

In summary, the gut microbiota of pwCF is distinct from that of healthy cohorts and several consistent alterations have been identified. The factors that shape the gut microbiota across age and location, and how these outcomes influence airway and other health outcomes, remain to be elucidated.

## MECHANISMS OF MICROBIAL INFLUENCE

### Direct fecal-oral transmission.

The correlation of lung health outcomes with diet, microbiota structure, and the prevalence of specific microbes raises the intriguing question of how gut-resident microbes might influence the lung and other organs distal from the intestine. There is emerging evidence to indicate that direct transmission of microbes between the gut and the lung is one important factor influencing the gut-lung axis (Table 1). The directionality of this interaction is not always clear. For example, pwCF have higher rates of Staphylococcus and Pseudomonas in the gut, but this could be either microbes seeding from the respiratory tract to the gut, or vice versa (62, 90). Longitudinal sequencing of URT and stool samples from infants with CF demonstrated a core microbiota shared between the URT and the gut (31, 32). These studies also found that 7 genera present in the URT were first present in the gut, indicating that these microbes are potentially seeded directly from the gut to the respiratory tract (31, 32). Two genera in the core microbiota are Streptococcus and *Veillonella*, both of which are increased in the lungs and intestinal tract of pwCF; some species of Streptococcus are also known CF lung pathogens. Many of the microbes identified are obligate anaerobes, which are not traditionally known as CF lung pathogens. Anaerobes appear to play a variety of conflicting roles in relation to CF lung disease; anaerobes are correlated with milder lung disease, but early colonization by anaerobes that can degrade mucins may also pave the way for later colonization by CF pathogens (91–94). These findings with anaerobes link to the climax-attack model of microbial community development in CF, which proposes that strict anaerobes play an important role in antibiotic resistance (95). In this model, an attack community of pathogenic microorganisms initially invades and causes a strong innate immune response and tissue scarring. Damaged tissue is then colonized by less-pathogenic microbes, including strict anaerobes, which are slower growing but more highly antibiotic resistant. The perpetuation of this cycle in the climax-attack model drives long-term airway remodeling in pwCF and could represent another example of how gut microbiota link to airway disease.

In addition to direct transmission of microbes, there is also evidence that nonmicrobial gut contents reach the airway (Fig. 1). Gastroesophageal reflux is highly prevalent in pwCF and microaspiration of gastric refluxate can promote the production of proinflammatory cytokines (96, 97). Caparrós-Martín et al. found that bile acid profiles of bronchoalveolar lavage fluid (BALF) from pwCF clustered into two distinct categories, with higher and lower bile acid (96). The higher bile acid cluster had a significant increase in some proinflammatory cytokines, but at the time of bile acid collection, structural deterioration was not different in the high and low bile acid clusters. However, bile acid abundance was predictive of lung outcomes during follow-up visits, where patients with higher bile acid had significantly more structural deterioration than patients with lower bile acid. Flynn et al. found lung microbiota taxa alterations specific to high or low bile acid concentrations, and that lung pathogen counts were positively associated with bile acid quantity (97). Thus, bile acid transfer to the airway represents a potential mechanism of gut-lung axis interaction, as bile acid has been correlated with lung inflammatory markers, pathogen abundance, and disease progression. It is possible that other microbial products, including proinflammatory pathogen-associated molecular patterns (PAMPs; e.g., LPS or flagella) and SCFAs, which are proinflammatory in the CF lung (in contrast to the intestine, where they are thought to be anti-inflammatory [98–100]), also directly modulate airway inflammation and disease (101–103), although there is scant evidence for these ideas in the CF literature.

### Inflammation and the intestinal microbiota.

CF exacerbations affect the entire body rather than having a strictly pulmonary target, as evidenced by the fact that serum calprotectin, a metal-sequestering protein secreted exclusively by neutrophils, is predictive of time to next exacerbation (104). Fecal calprotectin decreases after antibiotic treatment for exacerbation, indicating that multiorgan recovery occurs as well (105). Combined with the data described above indicating that microbiota composition is correlated with lung health outcomes, the inflammatory state of the intestine is of interest for both intestinal and lung health. Increased inflammation in the CF gut has been widely and consistently reported, and is typically measured as an increase in fecal calprotectin (54, 62, 106, 107). One exception is a proteomic analysis that did not find altered fecal calprotectin but did find increases in several other markers of inflammation (79). M2-pyruvate kinase (M2-PK), which is used as a marker of inflammation in people with IBD and colorectal cancer (108), is also increased in pwCF (54).

Children with CF who have high versus low intestinal inflammation have distinct microbiota compositions, and high inflammation has been associated with a Crohn’s-like dysbiosis (90). Intestinal inflammatory markers have also been correlated with specific taxonomic alterations in the gut microbiota of pwCF (24, 54, 62, 90). Notably, calprotectin is positively correlated with *Enterobacteriaceae* (24) or E. coli (34) relative abundance, but negatively correlated with *F. prausnitzii* and Lactobacillus paracasei (62). *Akkermansia*, a symbiont with known immunomodulatory properties that improve intestinal barrier function and promote tolerance of symbiotic microbes, was shown to be significantly higher in patients with normal levels of M2-PK (24). A study by Enaud et al. demonstrated increased Streptococcus and decreased *Bacteroides* and *F. prausnitzii* in a group of pwCF with high inflammation (90). Finally, a study of the colonic mucosa in pwCF revealed that gene expression of cancer-related genes is positively correlated with abundance of *Veillonella* and *Ruminococcaceae*, taxa that have previously been associated with colorectal cancer (87). It should be noted that while intestinal inflammation is consistently associated with intestinal microbiota alterations, there is minimal consensus across these studies as to which taxa are altered.

Importantly, it is unclear whether microbes drive inflammation or whether inflammation drives microbial dysbiosis (or both), although current evidence points toward a model where the changes in the CF intestinal environment select for a more proinflammatory gut microbiota. One compelling study hypothesizes that fecal fat selects for a proinflammatory microbiota, in particular increased abundance of E. coli (35). Furthermore, *Odoribacter* and *Bacteroides*, both of which are decreased in pwCF, have both been shown to reduce production of the proinflammatory cytokine IL-8 by gut epithelial cells (33, 109). Additionally, *Odoribacter* promoted secretion of the anti-inflammatory cytokine IL-10 by peripheral blood mononuclear cells (PBMCs) (109).

A small number of studies in CFTR knockout mice indicate that reducing microbial load in the small intestine, either by antibiotics or laxative treatments, can normalize inflammatory gene expression, as well as enterocyte maturation and host expression of genes for nutrient acquisition (110, 111). Furthermore, a 2016 *in vivo* study by Bazett et al. demonstrated that alteration of the gut microbiota via the antibiotic streptomycin can reduce airway hyperresponsiveness in mice, as well as alter airway T-lymphocyte subsets (56). Specifically, the mechanism of action may be through a reduction of proinflammatory IL-17^+^ γδ T cells. However, these investigators also observed an increase in Th17 (CD4^+^ IL-17^+^), CD8^+^ IL-17^+^, and CD8^+^ IFN-γ^+^ T cells, all of which would be hypothesized to contribute to a hyperresponsive environment. Finally, a study by Meeker et al. utilizing germfree CF mice demonstrated that increased systemic TH17 responsiveness in CF mouse mesenteric lymph nodes and spleen is driven by microbial colonization (36).

Taken together, the data above indicate that microbes can influence the local and systemic inflammatory state as well as lung hyperresponsiveness. An important open question is whether and how the altered intestinal inflammatory state affects lung or other health outcomes in a clinical context. While the lungs and intestines are distal, they are connected through a shared mucosal immune system, and immunologic cross talk is one of the primary mechanisms by which the gut is thought to influence the gut-lung axis (37). Given that pwCF have higher intestinal permeability (112–115), it is also possible that the intestinal inflammatory state influences the entire body through direct leakage of intestinal contents. Intestinal microbiota composition is associated with lung health outcomes; it is therefore reasonable to hypothesize that intestinal inflammatory state could influence health outcomes by one of the mechanisms described above. Few clinical studies have tried to assess this link, and the available evidence is conflicting (Table 4), with most studies either not directly assessing or finding no significant difference in ppFEV_1_ between groups with high and low intestinal inflammation (90, 116). No study has yet investigated whether intestinal inflammation is predictive of respiratory exacerbation rate. It is important to note that most current studies have focused on fecal calprotectin as a marker of intestinal inflammation. Given the clear gut-lung connection but apparent irrelevance of calprotectin in predicting this connection, it is important to consider that calprotectin may not be an appropriate measure of inflammatory status in this particular context.

**TABLE 4:** Associations between intestinal inflammation and health outcomes

Reference	Inflammatory marker(s)	Health outcomes	Results
Bruzzese et al., 2007 (166)		ppFEV_1_, BMI, respiratory exacerbations	*Lactobacillus* GG improved ppFEV1 and reduced pulmonary exacerbations.
Bruzzese et al., 2014 (106)	Calprotectin		*Lactobacillus* GG reduced calprotectin.
del Campo et al., 2014 (71)	Calprotectin	ppFEV_1_, BMI, cytokines	Probiotic treatment reduced calprotectin but does not improve ppFEV_1_, BMI, or other inflammatory markers.
Enaud et al., 2019 (90)	Calprotectin	ppFEV_1_, BMI	High intestinal inflammation was not associated with significant changes in BMI or ppFEV_1,_ but was associated with significantly more antibiotic exposure.
de Freitas et al., 2018 (62)	Calprotectin	BMI, ppFEV_1_	Antibiotic treatment was associated with higher stool calprotectin and lower BMI. The relationship between calprotectin and BMI or ppFEV_1_ was not assessed.
Coffey et al., 2019 (54)	Calprotectin, M2-PK	Growth, ppFEV_1_	Specific intestinal genera associate with intestinal inflammatory markers, growth z-scores, and ppFEV_1_. Correlation between outcomes and intestinal inflammatory markers was not directly assessed.
Dhaliwal et al., 2015 (116)	Calprotectin	Growth, ppFEV_1_	Inflammation was negatively associated with ht and wt but not ppFEV_1_.

### Microbial metabolites as potential drivers of the gut-lung axis.

Microbially produced metabolites are essential for both the development and maintenance of immune homeostasis. These metabolites can act locally in the intestine to alter cytokine production and/or immune cell programming (37, 117) or, as mentioned above, due to the increase of intestinal permeability in pwCF (112–115), may be able to enter the serum or tissues and act systemically (Fig. 1). Thus, metabolites derived from intestinal microbiota likely function via several mechanisms, including by tuning the immune response to act at a distance and influence lung health.

Microbial metabolites can be measured directly, or their production and degradation can be inferred through metagenomic or 16S rRNA sequencing. The most-well-studied of these metabolites are SCFAs, which are produced by microbial fermentation of dietary fiber. Acetate, propionate, and butyrate are present in stool at millimolar concentrations and are known to lower inflammation and intestinal permeability (98–100). SCFAs, particularly butyrate, also serve as an important energy source for colonic cells (98, 118). Finally, butyrate has been demonstrated to play an important role in the gut-lung axis in allergic asthma (119). For these reasons, there is significant interest in how microbial dysbiosis alters gut microbiota function and production of a variety of metabolites.

Metabolomic analysis of stool from pwCF has demonstrated that propionate and butyrate, but not acetate, are decreased (54, 67). Potential contributors to this decline include the decreased relative abundances of butyrate producers *Faecalibacterium* and *Roseburia* and propionate producers *Akkermansia* and *Bacteroides*. A proteomic analysis of stool found that two *F. prausnitzii* proteins involved in butyrate production were lower in pwCF (79). Metagenomic data has also demonstrated that catabolism pathways for both butyrate and propionate are increased in stool from pwCF (35, 54). Interestingly, there is a positive correlation between degradation of these SCFAs and increases in both calprotectin and fecal fat (35). Decreases in SCFA production may be exacerbated by the altered metabolism of microbes in the CF gut. For example, Miragoli et al. found a reduction in H_2_-consuming microorganisms, which could lead to increased hydrogen accumulation, where hydrogen inhibits normal fermentative reactions (120). *In vitro* work in fermentation systems by Wang et al. (60) demonstrated that CF stools had reduced capacity for acetate production and reduced microbial cross-feeding; additionally, a metagenomic analysis in this study showed reduced presence of *pta*, an essential gene in the acetate production pathway (60). While the findings of this study conflict with two metabolomic studies mentioned above that showed no change in stool acetate (54, 67), this work by Wang and colleagues is an important example of functional niches occupied by different species, as the authors determined that in healthy stools *Faecalibacterium* is the primary producer of SCFAs, whereas, in CF stools, *Clostridium sensu stricto* filled this role.

Similar to relative abundance data, microbiota functional profiles from pwCF converge with healthy individuals with age (35), but remain distinct from healthy individuals into adulthood (66). Metabolic alterations of the CF intestinal microbiota are not limited to SCFAs; significant differences in other microbial genetic pathways have also been detected in CF stool in both children and adults (35, 66, 121). For example, multiple studies have identified increases in virulence factors and xenobiotic degradation (66, 121). Increased exposure to antibiotics has likely contributed to an observed increase in capacity for xenobiotic degradation (66, 121), while increased virulence factors are likely due to increases in pathogenic microbes like E. coli.

Several studies have linked changes in metabolic pathways back to specific microbes. Vernocchi et al. demonstrated that CF patients have a different suite of microbes within the *Bifidobacterium*, *Lactobacillus*, *Faecalibacterium*, and *Eubacterium* genera, and a PiCRUSt analysis of these microbes predicted that their genomes would express different functions (121), including an increase in fat metabolism, loss of bile acid biosynthesis, and increased protein catabolism. Fouhy et al. found that changes in fatty acid metabolism were driven by *Ruminococcus*, *Clostridia*, *Enterococcus*, and *Eggerthella* (66). Increased E. faecalis is also a contributor to increases in lactose utilization, starch degradation, and antibiotic resistance. Interestingly, some genera that were not found to have altered relative abundance in this particular study, such as *Bacteroides*, still contributed to pathway alterations, potentially due to genetic differences in the colonizing strains (66).

Because different microbes can fulfill the same metabolic niche, identification of altered metabolic pathways have the potential to identify which microbes and metabolites actually contribute the most important changes to the CF gut microbiota. Alterations in SCFA production offer an interesting mechanistic hypothesis for regulation of the gut-lung axis in CF, but further investigation is needed to determine whether alterations of SCFAs, or of any of the other pathways identified, can influence health outcomes in the airway, and if so, how?

## DRIVERS OF MICROBIOTA ALTERATIONS

### CFTR genotype, disease severity, and multiorgan functionality.

Reduced CFTR function drives physiologic alterations in the gut, including thicker mucus, slower motility, and reduced pH. Evidence from multiple studies indicate a role for CFTR function in driving microbial dysbiosis in the CF gut (36, 67), and CFTR mutation may be a more important contributor than antibiotic exposure, birth mode, or breastfeeding (33). This finding is further supported by a study in CF mice, which have dysbiotic intestinal microbiota despite not being exposed to antibiotic treatments that are typical for pwCF (36, 56, 89). Furthermore, a study in germfree CF mice has demonstrated that when CF mice receive a fecal microbiota transplant from non-CF mice, the CF mice still have a significantly different microbiota from non-CF mice (36). Patient genotype can also influence microbiota structure (122), with more severe genotypes, such as F508del, having more extreme versions of the same dysbiosis. For example, patients with severe disease had further increased E. coli but lower *Faecalibacterium* and *Bifidobacterium* than patients with milder genotypes (122).

Lung complications account for the majority of CF-related mortality, and this review is primarily focused on the gut-lung axis for this reason. However, the gut microbiota influences full-body health and outcomes for other distal organs that can, in turn, influence gut health. For example, CFTR function is closely related to pancreatic function, prevalence of CF-related diabetes (CFRD), and incidence of CF liver cirrhosis (25, 123–125).

Pancreatic status is difficult to separate from CFTR genotype, as pancreatic status can be used as an indicator of CFTR dysfunctional severity (59). Of the few studies to examine the influence of pancreatic sufficiency on the intestinal microbiota, most have observed a modest influence (53, 59, 126). In a study by Nielsen et al., pwCF with pancreatic sufficiency trend toward higher alpha-diversity and have significant alterations in some taxa that are typically intermediate relative to the healthy and pancreatic insufficient CF cohorts. For example, *Lachnospiraceae incertae sedis* and *Erysipelotrichaceae* are reduced overall in pwCF, but are further reduced in the pancreatic insufficient group (59). Interestingly, the genus *Oscillibacter* is in higher relative abundance in a pancreatic sufficient group of pwCF compared to both pancreatic insufficient pwCF and the healthy cohort. Burke et al. observed significant changes as well, but in different taxa; *Ruminococcus* and *Anaerotruncus* are increased in a pancreatic sufficient group compared to pancreatic insufficient pwCF (53). Pope et al. found significant increases in *Proteobacteria* and *Bacteroidetes* and significantly decreased *Actinobacteria* in a pancreatic sufficient group (126). Of note, the milder mutations that lead to CF with pancreatic sufficiency are rarer than severe mutations, and the largest of these studies included only 12 individuals in the pancreatic sufficient group, which likely contributes to the lack of consistency and significant observable alterations.

Bile acids are likely important in shaping the gut microbiota, but the specific effects of altered bile acids have not been examined in pwCF. Bile, produced by the liver, is higher in the stool of pwCF, but the bile is more viscous, has lower hydrophobicity, and reduced alkalinity (123, 127). Bile acids are typically deconjugated by bacteria in the intestine, and reduced *Bacteroides* may contribute to lower levels of bile salt deconjugation observed in pwCF (127). This example highlights how mutations in CFTR may modulate the gut microbiota, but this altered gut microbiota can also, in turn, further modulate the intestinal environment. Finally, as mentioned above, the altered bile in the gut of pwCF could impact airway function directly via physical transfer to the airway (Fig. 1).

Approximately 30% of pwCF also have cystic fibrosis-associated liver disease (CFLD), which is the third highest cause of death, after lung disease and lung transplant complications (123). The mechanism of liver disease development in CF is not fully understood and there are competing hypotheses which will not be discussed in depth here. Briefly, one hypothesis is that thick, viscous bile and bile duct blockages cause direct damage to hepatocytes followed by induction of a proinflammatory environment and further damage (123). An alternative hypothesis proposed by Flass et al. suggests the existence of a gut-pancreas axis where the increase in microbial pathogens in the gut, combined with increased gut permeability, drive liver damage and cirrhosis (112). An example of microbe-driven liver disease can be seen in alcoholic liver disease, where the presence of intestinal Enterococcus faecalis strains producing cytolysin toxin exacerbates alcohol-induced liver damage (129). In comparing the intestinal microbiota of a cohort of pwCF with and without cirrhosis, Flass et al. found that patients with cirrhosis had slower small bowel transit time and an altered gut microbiota relative to patients without liver disease. In particular, *Bacteroides* were decreased, and *Clostridium* were increased in the cirrhosis cohort, following the standard pattern of alterations for pwCF, but more extreme for those individuals with CF liver disease. Additionally, higher *Bacteroides* was associated with less macroscopic intestinal injury, while the presence of *Clostridium* was associated with more such injury. However, cirrhosis was not associated with increases in calprotectin or intestinal permeability, which are other markers of intestinal inflammation. The impact of gut-driven liver disease on airway health is an open question.

### Antibiotics.

People with CF receive frequent antibiotic treatments, and this is often assumed to be a major contributor to intestinal dysbiosis. This assumption is reasonable given the known impacts of antibiotics on gut microbiota structure (130, 131). However, evidence is mixed on how strongly antibiotics influence the CF gut microbiota, likely due to the fact that the CFTR mutation causes many intestinal physiological alterations in the absence of antibiotic therapy, as described above.

Microbiota alterations and inflammatory markers have been shown to worsen after exposure to antibiotics in some studies (62, 106). Antibiotic treatments also decreased alpha-diversity and relative abundance of the beneficial symbionts *Bifidobacterium* and *Bacteroides* (53, 58, 62, 63, 106), as well as increasing *Enterococcus* (63). De Freitas et al. found that the only significant difference in microbiota structure due to antibiotic exposure was decreased *Bifidobacterium* in the antibiotic-exposed group (62). Burke et al. examined the effects of intravenous (i.v.), nonmacrolide antibiotics and orally administered macrolide antibiotics separately and saw that i.v. antibiotics significantly correlated with decreased alpha-diversity but orally administered macrolide antibiotics did not (53). However, pwCF with recent macrolide antibiotic treatment had significant alterations in several taxa, including decreased *Akkermansia* and *Bifidobacterium*. Of the studies described, Burke et al. is the largest and also identifies the most alterations, indicating that the influence of antibiotics may be nuanced and specific to the type of antibiotic treatment (53).

Unexpectedly, some studies show the potential for positive impacts of antibiotics on the CF microbiota. Fecal calprotectin is decreased at the end of an exacerbation after treatment with antibiotics (105). Additionally, treatment with azithromycin has been associated with weight gain when administered to children with pulmonary diseases (132–135). However, azithromycin is also associated with decreases in the beneficial symbionts *Bacteroides* and *Bifidobacterium* in wild-type Sprague-Dawley rats (136). Interestingly, azithromycin treatment also decreased *Enterobacteriaceae* (136), an outcome that is not recapitulated in data from pwCF, who frequently have increased relative abundance of *Enterobacteriaceae* (35, 54). In mice, administration of the antibiotics ciprofloxacin and metronidazole increased microbial diversity in one study (89), while streptomycin treatment reduced airway hyper responsiveness, SIBO, and inflammatory profiles in another (56).

The impact of antibiotic treatment on the gut microbiota in CF remains an open question and is not likely to have a simple summary; antibiotic effects appear nuanced depending on the antibiotic class, route of delivery, and likely many additional factors. Furthermore, two recent studies of the airway microbiota and its response to antibiotic treatment align with a somewhat open understanding of antibiotic impacts on the gut microbiota. That is, exposure of the airway microbiota to high levels of aerosol antibiotics found minimal impact on CF major airway pathogens with some observed reductions in less abundant microbes (137, 138). Thus, sweeping assumptions about the impacts of antibiotics on microbiota should be made with some caution.

### Diet and nutrition.

Nutrition is nearly impossible to extricate from the gut-lung axis, as demonstrated by early CF gut microbiota studies, which observed that breastfeeding could lead to changes in the respiratory microbiota, in addition to the intestinal microbiota, and could be correlated with time to initial exacerbation (31, 32). Furthermore, gut microbial dysbiosis has been associated with linear growth failure for children with CF, with more extreme dysbiosis correlated with lower length in infants (61). The highest priority for children with CF is to ensure sufficient weight gain and growth. However, current dietary recommendations may have unforeseen consequences on the gut microbiota. This section will detail those potential consequences and describes current research on dietary interventions that can be made without compromising overall nutrition.

Due to fat malabsorption and difficulty gaining weight, many pwCF maintain a high-fat diet (139). High-fat diets are known to modulate the microbiota in people without CF, including decreasing microbiota diversity and increasing markers of inflammation (45, 140). A high-fat diet can also reduce the amount of SCFAs produced by the gut microbiota and, over time, reduce the abundance of SCFA-producing symbionts (45). The combination of high-fat diet and fat malabsorption may be one of the primary drivers of microbial alterations and inflammation in the intestines of pwCF (35). A systematic review by Wolters et al. found that while a high-fat diet induces undesirable changes in the microbiota, polyunsaturated fatty acids (PUFAs) such as omega-3 do not have a negative impact (140). Interestingly, dietary intake of omega-3 has been associated with reduced cough and wheeze in asthma and COPD, as well as reduced serum inflammatory markers in COPD (141–144). However, life history and intake of proinflammatory fatty acids, such as omega-6, are also important factors driving these outcomes (141). A recent systematic review of omega-3 supplementation for pwCF found mixed evidence of omega-3 supplementation on ppFEV_1_ outcomes and the authors emphasized the need for additional randomized-control trials due to the low quality of the current evidence (145).

In mice, even a short-term high-fat diet altered both the microbiota and immunologic expression, causing dense colonization of the normally microbe-free intervillous zone of the small intestine and microbiota alterations reminiscent of CFTR defect, including increases in *Proteobacteria* and decreases in *Bacteroidetes* (146). This high-fat diet also caused decreases in CFTR gene and protein levels and antimicrobial peptide expression, demonstrating that a high-fat diet may exacerbate CF intestinal complications. Interestingly, these changes were largely reversible by treatment with rosiglitazone, an agonist of the peroxisome proliferator-activated receptor (PPAR-γ). Furthermore, rosiglitazone had positive effects in CFTR^−/−^ mice, including increased bicarbonate secretion and reduction of mucus in the intestine (147). Early promising results in mice with rosiglitazone prompted speculation that it could be used in pwCF, but due to severe side effects with long-term use, rosiglitazone has been removed from the market in several countries (148–150). However, PPAR-γ remains an interesting potential target for improving CF outcomes.

In contrast to fat, dietary fiber has been shown to reduce inflammation and improve lung outcomes in respiratory diseases such as asthma and COPD (45). These improvements are likely due to expansion of SCFA-producing gut symbionts. cwCF who had higher fiber intake also had fewer abdominal symptoms (151). No study has yet looked at the effects of fiber intake on the gut-lung axis or the microbiota structure in a CF cohort. However, this type of study could be complicated because only cwCF with normal weight gain are recommended to increase fiber intake, due to the risk of increased fiber reducing overall calorie consumption (151).

pwCF have difficulty absorbing fat-soluble vitamins, such as vitamin D. Studies have shown that higher serum vitamin D is significantly associated with fewer annual pulmonary exacerbations (55, 152). Vitamin D also has the capacity to shape the microbiome, with vitamin D deficiency associated with higher *Gammaproteobacteria* and lower *Bacteroides* (153). Supplementation with vitamin D significantly altered both the gut and the lung microbiomes for pwCF, and notably led to a decrease in *Veillonella* and *Erysipelotrichaceae* in the gut, as well as lower Staphylococcus and *Corynebacterium* species in the lung. Dietary intake of flavonoids and other micronutrients have also been associated with gut microbiota structure for pwCF (154, 155).

In summary, diet is both a contributor to and potentially a partial solution for dysbiosis for pwCF. It is unclear whether it is possible to adjust diets for pwCF to increase fiber, as this may only be appropriate for patients with mild disease or on the newest medications. Adjusting the type of fat intake may be appropriate for a broader proportion of pwCF, but evidence of positive outcomes from this approach is not strong. Increased intake of vitamin D, micronutrients, or specific fatty acids may also have positive influences on both the gut microbiota and lung health outcomes. However, more studies are needed to determine whether these types of changes would have meaningful impacts in the context of CF.

## REMEDIATING THE CF MICROBIOTA

### Modulators.

CFTR modulators have had significant positive impacts on the life span and lung health outcomes of pwCF (156, 157). While the intestinal microbiota is not an intended target, it has the potential to be impacted by modulator treatments when CFTR becomes more active and alters the gut microenvironment. Several studies have examined the impacts of modulator therapies on the airway microbiota, with apparently modest improvements in pathogen burden and changes in lung microbiota in some but not all studies (158–160). However, there are two studies to date that look specifically at the effects of modulators on the gut microbiota of both children and adults with CF (24, 126). These studies examine the effects of ivacaftor and ivacaftor/lumacaftor. Ivacaftor, approved for the mutation G551D and other mutations of similar function, increases intestinal pH to near-healthy values (26). Ivacaftor treatment was shown to decrease fecal calprotectin in a small CF cohort, as well as to significantly increase the relative abundance of *Akkermansia* (24). An exploratory analysis by Pope et al. examined changes in the intestinal microbiota of both pancreatic sufficient and insufficient patients upon initiation of ivacaftor and ivacaftor/lumacaftor, respectively (126). This study did not find any significant changes after modulator initiation for alpha-diversity, microbiota composition, or fecal fat in either group. However, the pancreatic sufficient group trended toward decreased fecal fat, and the microbiota structure also trended toward that of the pancreatic sufficient group after treatment with modulator therapy. Interestingly, in the pancreatic sufficient group, antibiotics had a greater impact on the intestinal microbiome than modulator therapy, a result consistent with that of a lung microbiota study on the effects of ivacaftor (159). A large upcoming study, PROMISE, will examine many effects of modulator therapies, including gut microbiota alterations (161). The PROMISE study is particularly important, as it will be the first to examine the effects of the most recently approved modulator combination therapy, elexacaftor/tezacaftor/ivacaftor (trikafta).

### Pre-, syn-, and probiotics.

The clinical connection between the intestinal microbiota and lung health outcomes has sparked interest in probiotic interventions, as well as the application of more novel pre- and synbiotics. Despite this interest, CF-specific studies are sparse and there is a need for more randomized clinical trials (162). Probiotic treatment is defined here as the administration of live microbes with putative health benefits. The most consistent finding from probiotic clinical trials in pwCF is a reduction in respiratory exacerbations (57, 163, 164), although this result is not consistent across all studies (165). Notably, probiotic treatments can be formulated with one or more microbial strains, and probiotic composition varies between CF clinical trials. A promising result from Jafari et al. (57) demonstrated significant improvement in the short term for both pulmonary exacerbations and quality of life score for cwCF receiving a commercial probiotic, indicating that any probiotic supplementation may need to be continuous to have the desired effect (57). Additional short-term experiments demonstrated the capacity of *Lactobacillus* GG to reduce fecal calprotectin (107), as well as to significantly increase the relative abundance of the symbiont *Bacteroides* (106). *Lactobacillus* GG (LGG) administration has also been shown to positively impact clinical outcomes for pwCF infected with Pseudomonas (166). While actively receiving LGG, pwCF demonstrated decreased respiratory exacerbations and hospitalizations but increased body weight and ppFEV_1_ (166). Another *Lactobacillus*, Lactobacillus reuteri, decreased pulmonary exacerbations and URT infections, but not fecal calprotectin, in one clinical trial (163). However, in a separate trial, L. reuteri significantly decreased calprotectin, increased GI comfort scores (GIQIL), increased microbial alpha-diversity, and showed a very modest, nonsignificant trend toward reduction of fecal proinflammatory cytokines (71). It is notable that while L. reuteri is a commercially available probiotic and is not itself reduced in pwCF, it was still able to promote growth of symbionts that are reduced in pwCF. This effect was also seen in a mouse model of CF where Lactobacillus acidophilus restores *Bacteroides* and *Bifidobacterium* after reduction by azithromycin, a common CF antibiotic (136). How such probiotic microbes help restore endogenous microbes is unclear.

Several of the probiotic clinical trials mentioned above use the crossover method, allowing individual patients to serve as their own controls (71, 165, 166). This approach can be powerful for studies of pwCF, where patient populations are small and interindividual variability is high. All of these studies were done in stable cohorts; this makes sense because of the confounding effects of antibiotics, as well as higher risk in medically unstable cohorts. However, if probiotic treatment is ever widely implemented for pwCF, it is possible that beneficial effects would be larger in patients with more severe disease.

Prebiotics are carbon sources that are not digestible by humans but are intended to promote the growth of beneficial gut symbionts. Prebiotics have remained relatively unexplored in pwCF. An *in vitro* study examining the effects of prebiotic high-amylose maize starch (HAMS) showed that the CF gut microbiota has the capacity to respond to HAMS, albeit with a different suite of microbes and to a lesser extent than detected in non-CF stools (60). Synbiotic supplementation is the combination of pre- and probiotics in the same treatment. Only one study, reported in two separate publications, has been conducted using synbiotics in pwCF (167, 168). This study saw no significant impact of 6 months of synbiotic treatment on quality-of-life scores, pulmonary exacerbations, hospitalizations, or ppFEV_1_. Overall, there is fertile ground for exploring the role of pre-, syn-, and probiotics in pwCF. Furthermore, in the context of CF, probiotic studies replacing the microbes missing, particularly in young pwCF, remain to be performed.

## MOVING FORWARD

### The gut-lung axis: an experimental wish list.

Many open questions regarding the gut-lung axis in CF remain, both from a basic science as well as a clinical perspective. Clinical sampling of inflammation and microbial dysbiosis in stool are the best-studied aspects of the CF intestinal milieu. However, more data are needed on dysbiosis during important developmental windows and how particular timing or structure of dysbiosis may alter lung health outcomes later in life. Furthermore, metagenomic studies have the potential to fill two important knowledge gaps: changes in microbiota functional potential and alterations in nonbacterial components of the microbiota. Notably, while the intestinal fungal mycobiome has been demonstrated to influence airway outcomes (169), no studies to date have examined this idea in the context of CF. Due to the rare nature of CF, many clinical microbiota studies of pwCF are small. Meta-analysis of available data is a highly appealing but currently underutilized method for understanding broadly consistent trends in the CF gut microbiota. However, it is promising that many recent studies have made their raw sequencing data publicly available (33, 53, 61, 63, 66, 67), allowing meta-analyses of these data.

There are very few basic microbiological and immunological studies of the gut-lung axis in pwCF, and these will be essential for understanding the mechanism of gut-lung cross talk. Mouse models have been used frequently in studies of the asthma gut-lung axis and may serve as a model for how to conduct mechanistic immunologic CF gut-lung axis studies (37–48). Mouse models of CF were not originally designed for intestinal studies, and several models contain either a genetic gut correction where wild-type CFTR is expressed in the intestinal epithelium, or mice are required to be kept on a special, microbiota-altering diet. These choices, while practical for maintaining mouse viability, make many mouse models of CF inappropriate for intestinal microbiota studies. Fortunately, mouse models do exist that do not require gut correction and can be maintained on a standard diet. A caveat to these models is that the CF gut microbiota structure appears to be model dependent (170).

An early study by Bazett et al. may serve as a model for future mechanistic work in the CF gut-lung axis; they combined microbiota alterations through antibiotic treatment with measures of airway outcomes and inflammation (56). While a promising early study, antibiotic treatment does not offer a nuanced approach to determine the effects of specific microbes due to the broad impacts of systemic antibiotic treatment on a variety of intestinal microbes as well as, potentially, the native airway microbiota. Future studies on alterations of specific intestinal microbes will be important for defining gut-lung axis interactions.

Microbiota structure, as determined by sequencing of stool samples, can be used to inform areas of interest in microbiology and may serve as a jumping-off point for more mechanistic microbial studies. Changes in the CF gut environment are likely to affect not only which microbes are present but also the metabolic potential and genomic content of the microbes that do thrive. Genetic and metabolic adaptation of opportunistic pathogens to the CF lung environment has been extensively documented (171–173), but very few studies have examined such features in the CF gut. One study demonstrated that E. coli from the CF gut have adapted to the high fat environment, as evidenced by increased growth rate and reduced stress response with glycerol as a sole carbon source (174). The ability of E. coli to adapt to this high-fat environment may also partially explain its increased relative abundance in pwCF. This finding highlights the importance of environmental conditions in shaping the CF gut microbiota. Because gut symbionts are frequently metabolically interdependent, small alterations are likely to have ripple effects through disruption of cross-feeding. For example, cocorrelations can be seen between specific genera in CF microbiota, including a positive cocorrelation of *Bacteroides* and *L. paracasei*, and a negative cocorrelation between *Veillonella* and Eubacterium rectale (62). A valuable tool in mechanistic CF microbiology airway research has been the development of artificial sputum medium (ASM), which is used to mimic the CF sputum environment. We suggest that an *in vitro* growth medium reflecting the intestinal milieu (175), analogous to ASM, will serve to further push CF intestinal microbiota studies forward, especially for studies of microbial interactions in the context of the CF intestinal environment. Overall, understanding the mechanisms of microbial adaptation in the CF intestine is an area ripe for future study.
